# Living apart together: reflections on bioethics, global inequality and social justice

**DOI:** 10.1186/1747-5341-3-25

**Published:** 2008-12-07

**Authors:** Stuart Rennie, Bavon Mupenda

**Affiliations:** 1 Departments of Dental Ecology, Social Medicine and Health Policy and Management, University of North Carolina at Chapel Hill, NC, USA; 2 Centre Interdisciplinaire de Bioéthique pour L'Afrique Francophone (CIBAF), Kinshasa School of Public Health, Kinshasa, Democratic Republic of Congo

## Abstract

Significant inequalities in health between and within countries have been measured over the past decades. Although these inequalities, as well as attempts to improve sub-standard health, raise profound issues of social justice and the right to health, those working in the field of bioethics have historically tended to devote greater attention to ethical issues raised by new, cutting-edge biotechnologies such as life-support cessation, genomics, stem cell research or face transplantation. This suggests that bioethics research and scholarship may revolve around issues that, while fascinating and important, currently affect only a small minority of the world's population. In this article, we examine the accusation that bioethics is largely dominated by Anglophone and industrialized world interests, and explore what kinds of positive contributions a 'bioethics from below' (as Paul Farmer calls it) can make to the field of bioethics in general. As our guide in this exploration, we make use of some experiences and lessons learned in our collaborative bioethics project in the Democratic Republic of Congo, *Building Bioethics Capacity and Justice in Health*. We conclude that while there is some evidence of increased attention to bioethical challenges in developing countries, this development should be further cultivated, because it could help expand the horizons of the field and enhance its social relevance wherever it is practiced.

## Introduction: bioethics and inequality in health

There are vast differences in health between low-income, middle-income and high income countries around the world, as well as significant differences in health within these countries. Epidemiologists, health economists and health policy makers typically express global health inequalities in the form of differences between country health (and health proxy) indicators such as life-expectancy, maternal and child mortality, and average per capita income. For example, there is more than a 2-fold difference in life expectancy between the top three countries (Japan, 82.3 years; Hong Kong, China, 81.9 years; Iceland, 81.5 years) and the bottom three (Zambia, 40.5; Sierra Leone, 40.7; Zimbabwe, 40.9) [1]. In terms of child mortality (one year and younger), commonly considered an indicator of country health or development, the worst affected countries have rates more than 90 times higher than those least affected. In regard to maternal mortality, the lifetime risk of pregnancy-related death in Malawi is 1 in 7, as compared with 1 in 2800 in industrialized nations[2]. As is well known, income is inversely related to infant mortality and a host of other health indicators. There is a 400-fold increase between average per capital income among the richest and poorest countries, and although average income overall in the world has increased in the past few decades, the differences at the outer ends of the global income index have been widening significantly[3,4]. In addition, research has indicated that not only does absolute poverty have a strongly negative impact on health, but the greater the disparity between rich and poor in a given society, the worse the health of the 'less fortunate' in such societies tends to be. Inequality, as Norman Daniels puts it, is bad for your health [5,6].

Given the importance of health as a human value, and the traditional aspiration of bioethics to articulate universal principles, one might predict that ethical issues related to these appalling global disparities would feature very prominently in bioethics scholarship and research. However, some commentators claim that (a) mainstream bioethics research and scholarship is marked by excessive attention on bioethical issues largely affecting the world's more affluent countries and (b) that in the light of global realities, the predominantly 'first-world' agenda of bioethics should change. Leigh Turner, for example, writes that many of the questions that bioethicists address (such as face transplantation, cessation of life-support, or prenatal genetic diagnosis) are only intelligible in the contexts of wealthy developed nations, and these topics appear 'trivial' when compared to the kinds of health issues faced by people in impoverished, developing countries. Unless bioethics broadens its agenda, Turner writes, it risks becoming a form of entertainment[7,8]. In a similar vein, Paul Farmer writes that mainstream bioethics has largely been focused on issues of personal autonomy in regard to new developments in biotechnology, rather than the problems of social justice arising from the growing (health) gap between the world's rich and poor communities[9]. Steven Miles writes that the 'soul of bioethics' has been rendered unhealthy partly by its tendency to engage more with issues of assisted reproduction and gene therapy than with the growing number of medically uninsured in America, minority and migrant health, the links between health and human rights, or the political and economic barriers preventing developing countries from gaining greater access to essential medicines[10].

Does bioethics, as a field of research and scholarship, concentrate too much on 'problems of affluence' while neglecting the bioethical problems prevalent in resource-poor settings? And if so, should the agenda of bioethics be broadened, and in what ways? In attempting to answer the first question, it should be noted that there is currently little empirical data on what topics are studied by bioethicists or trends in bioethics scholarship over time. Borry et. al. [11,12] have studied authorship of bioethics publications, and have concluded that peer-reviewed bioethics journals describing themselves as 'international' largely publish articles by authors from Anglophone developed countries, particularly the United States, United Kingdom, Australia and Canada. However, as Borry et. al. admit, these findings do not in themselves show that the content of bioethics research and scholarship is biased towards the concerns, interests and perspectives of more affluent nations, though it is plausible that the social, cultural and class origins of bioethicists might significantly affect topic selection and directions of scholarship and research. In a recent study of trends in bioethics topics by Cohen et. al[13], the authors suggest that 'favored subject matter' in bioethics varies significantly over time due to legal controversies, discussion saturation and epidemiological importance. One striking finding of this study was that the number of publications on 'AIDS and ethics' rose from 16 during the period 1980–84 to a peak of 793 in the period 1990–94, and then sharply declined to 197 between 2000 and 2004. As the authors note, the interest in this topic among bioethicists in this case is strongly related to the epidemiology of HIV/AIDS in the United States. With increased public health surveillance and prevention efforts, the advent of (and access to) effective anti-retroviral treatment and the virtual disappearance of mother-to-child transmission of HIV through prenatal testing policies, the prevalence of HIV/AIDS dropped dramatically in the early 1990's, and the interest of bioethicists in the topic of AIDS and ethics seems to have declined with it. However, during this same period there were many millions of new HIV infections and AIDS-related deaths worldwide, with widespread and devastating economic, political and social effects. This suggests that HIV/AIDS was of significant interest and concern to the mainstream bioethics community when it significantly threatened the developed world, but is a considerably less compelling bioethics topic when HIV/AIDS affects poorer countries elsewhere.

The plausibility of 'first-world bias' in bioethics is further strengthened by relatively easily accessible data regarding the agencies who fund the bulk of bioethics research, the location of the vast majority of bioethics centers or publishing houses of bioethical journals[14]. It is probably safe to claim that most bioethicists originate from and/or work in developed countries. On the other hand, there is currently no firm data on origins, education or location of bioethicists, and the issue is further complicated by the fact that those who deal with ethical issues arising from health research, policy or practice may not self-identify as 'bioethicists' or publish their work in mainstream bioethics journals. In summary, while there is some credible evidence in favor of a current 'first-world bias' in bioethics, more empirical research should be conducted to evaluate the claims of bias and parochialism that continue to be made.

Indeed, there are signs that current global situation regarding bioethics is more complex than is sometimes depicted in 'first-world bias' claims. For example, in the past decade, there has been a sharp increase in the number of sophisticated biomedical institutions established in India, partly a response to the demand for medical tourism and the economic incentive to host large-scale clinical trials. Researchers and physicians working in these institutions are increasingly faced with standard 'first-world' bioethics issues such as cessation of life-support and death criteria in view of organ transplantation. In pockets of even the poorest countries, such bioethics issues are not trivial, and are likely to increase in relevance as the health standards of developing countries rise. The situation is similar with the bioethical issues surrounding assisted reproductive technologies as these become more accessible in resource-poor settings[15]. To further complicate the picture, controversial biomedical research on mother-to-child HIV transmission in Africa and Asia during the 1990's raised the profile of bioethics issues in developing countries in a number of important ways. The controversy increased attention on the issue of the appropriateness of placebo controlled trials in general when conducting clinical trials in poor countries, the content and role of international declarations protecting research participants, and the meaning of exploitation in the context of international research. The controversy was also the likely origin of the Nuffield Council on Bioethics' report *The Ethics of Research Related to Healthcare in Developing Countries *(2001), the Wellcome Trust's initiative to fund bioethics research in developing countries (started in 2002), and the International Research Ethics Education and Curriculum Development Awards offered by the Fogarty International Center at the National Institutes of Health (launched in 2000), which is responsible for training health professionals in bioethics and research ethics relevant to developing world contexts at 18 institutions worldwide. The European and Developing Countries Clinical Trials Partnership (EDCTP) has offered funds for establishing ethics review committees and bioethics educational programs in sub-Saharan Africa. *Developing World Bioethics*, a peer-reviewed journal launched in 2001, is devoted entirely to bioethics issues relevant to resource-poor settings and has become an important target journal for those working in this field. There is increasing talk of African[16-18], Muslim[19,20] or Buddhist[21,22] bioethics, and predictably, renewed challenges to the idea of universal bioethics principles applicable to all cultural contexts [23-25].

In short, while developed world topics, institutions and authors still tend to be predominant, the global bioethics landscape is slowly changing. What then are some key concerns that tend to be marginalized within the mainstream bioethics community but are more prominent in developing world contexts? We will explore this question through the prism of a collaborative bioethics program we have helped establish in Kinshasa, Democratic Republic of Congo.

### Centre Interdisciplinaire de Bioéthique pour L'Afrique Francophone (CIBAF) at the Kinshasa School of Public Health

The Democratic Republic of Congo (DRC) covers the largest geographical area (> 900,000 miles) and has the largest population (approximately 63 million) of all Francophone African countries, and is the second largest French-speaking country in the world. Its capital city, Kinshasa, is estimated to have a population of 8.9 million, making it the second largest city in sub-Saharan Africa, and the third largest city on the African continent after Lagos and Cairo. This former Belgian colony is economically and politically crucial to the sub-Saharan African region, but is only slowly recovering from decades of political oppression and mismanagement, violent civil conflict, and economic exploitation of its natural resources. The legacy has left many essential sectors, particularly education and medicine, in a state of disarray[26].

In 2004, the University of North Carolina-Chapel Hill, the University of Louvain (Belgium) and the Kinshasa School of Public Health applied for what was then called an *International Bioethics Education and Career Development Award *from the Fogarty International Center at the National Institutes of Health. The stated main purposes of the grant are (a) to improve the quality of international research ethics training (i.e. develop courses on bioethics and research ethics issues affecting resource-poor countries) and (b) to support advanced training of health care and other professionals from resource-poor countries, in order to improve ethical review of biomedical or public health research conducted in such settings. In our application for this grant, we proposed to train a small core of Congolese scholars in Belgium and/or the United States, who would establish and manage a center for bioethics at the Kinshasa School of Public Health on their return to the DRC. The center, later named the *Centre Interdisciplinaire de Bioethique pour L'Afrique Francophone *(CIBAF) was conceived as a place for research ethics and bioethics research, scholarship, education and consultation, focusing in particular on ethical issues faced in biomedicine and public health research, policy and practice in sub-Saharan Francophone Africa. The Fogarty project we proposed, entitled 'Strengthening Bioethics Capacity and Justice in Health', was approved for initial funding in 2004 and renewed funding in 2008.

Why strengthen bioethics and research ethics capacity in the DRC, given its troubled social, economic and political context? There have been some sharp criticisms of Fogarty bioethics projects and similar programs, namely that bioethics training of professionals from developing countries constitutes 'ideological transfer' of Western values, and the hidden agenda of the program is to facilitate US federally funded biomedical and public health research in resource-poor countries [27]. In our experience, these criticisms have limited application and relevance. 'Ideological transfer' tends to fade in the face of local values, realities and practical constraints, and we have managed to ensure that medical ethics and public health ethics – not just research ethics – retains a prominent role within CIBAF activities. For us, the bottom line is that – often in the face of tremendous challenges – medicine, public health interventions and biomedical research are conducted in Francophone African countries, complex ethical issues regularly arise from them, and explicit discussion of these issues is still rare in medical, in popular media, among NGOs or in local communities and other stakeholders. CIBAF takes a social justice perspective on these issues, befitting a context where the sub-standard health of the vast majority is clearly linked to man-made historical, social, cultural, economic and political forces. In the DR Congo, it is clear that many local bioethics and research ethics issues are ultimately rooted in unjust forms of inequality, and this consideration undermines any possibility for local bioethics to remain 'politically neutral'. Bioethical reflection on the problems stemming from health inequalities, including efforts to overcome them, is itself a form of political commentary.

### Ethical challenges raised by the struggle to improve health in resource-poor countries

In order to offer some examples of these ethical challenges, it is first important to ask: what are some of the most important ways of reducing global health inequalities? In what follows, we will briefly discuss what we see as prominent ethical challenges in five important and interrelated approaches to reducing global inequalities by improving health in resource-poor countries: (1) global health research, (2) implementation of tested health interventions, (3) changing of health policies, (4) strengthening of health care infrastructure, and (5) tackling upstream forces impacting on health.

#### Global health research

The general goal of clinical and public health research is to produce new, reliable information which could be used to improve the health conditions of individuals and/or populations. Prevention of mother-to-child HIV transmission research in the 1990's vividly demonstrated how pursuit of this worthwhile goal can raise ethical controversies in low-income countries[28,29]. The University of North Carolina at Chapel Hill and the Kinshasa School of Public Health currently conducts operational research on effective and appropriate provision of anti-retroviral treatment to HIV-positive persons in Kinshasa. Operational research – sometimes called implementation research – is considered a crucial preliminary stage in the process of integrating health interventions effectively and sustainably into health systems[30]. However, when the researchers wanted to involve HIV-positive minors (less than 18 years old) in their study, they ran into an ethical and regulatory quandary. On the one hand, local physicians informed the research team that minors are rarely told their HIV status by their parents or doctors. On the other hand, US regulations state that Institutional Review Boards have the discretion to require assent from minors when they participate in research. How could meaningful assent be obtained from minor participants – some of whom were already in their early and mid-teens – for HIV-related research without thereby disclosing their HIV-status to them? And is assent considered culturally appropriate by parents, guardians and local health care providers? How does the concept of assent – originating in the idea of the children's rights – relate to local conceptions of the relationship between parents and children in the context of medical decision-making? The involvement of HIV-positive children and adolescents in this operational research study would be undoubtedly beneficial for the participants, since those who need it would be provided with treatment known to be effective and to which only a small minority of children (or adults) currently have access in the Democratic Republic of Congo. While involving HIV-positive children in this research raises problems from an ethical and regulatory perspective, not involving them would be akin to the withholding of known effective treatment.

In response to this problem, qualitative research on issues surrounding pediatric assent and disclosure in HIV-related research was conducted among parents, guardians, physicians and young HIV-positive adults. The results of this research are or will be published elsewhere [31,32], but the main findings were that most youth interviewed believed minors participating in HIV-related research should be informed of their HIV-positive status, while parents/caregivers had varied perspectives on if and when HIV status should be disclosed to minors during research participation. The age of the youth influenced parents'/caregivers' responses, and disclosure to adolescents was more frequently supported than disclosure to children. Several parents/caregivers suggested that minors should never be told their HIV-positive status when participating in HIV-related research regardless of their age. The implications of these results for policy-making on pediatric assent and disclosure in HIV-related research and clinical practice were discussed in a workshop among a number of local stakeholders, including members of CIBAF, the Ministry of Health, the National AIDS Control Program and local health NGOs. In the case of the ongoing operational research, it was decided while assent should not be a requirement for the children's involvement in the research, disclosure of HIV status should be regarded as an ultimate goal for all HIV-positive youth (particularly as they become sexually active), and a gradual and individualized process of disclosure should be initiated for each child involving parents, physicians and psychosocial assistants. Our point here is not so much to weigh in on this particular case, but to give a taste of the kinds of scientific, cultural and ethical controversies that are part and parcel of health research in low-income countries. Of all ways of reducing global health inequalities, health research receives the most bioethical attention[33].

#### Implementation of tested interventions

The fact that many health interventions, already shown to be effective, have not been implemented in many low-income countries is itself a matter of (longstanding) serious ethical concern[34]. Millions of deaths and disabilities in these countries occur due to conditions we already know how to prevent or treat. But when one tries to buck this trend, new ethical challenges emerge. For example, there have recently been global initiatives (such as PEPFAR and the Global Fund) to increase access to AIDS treatment in low-income countries. Programs funded by such initiatives typically offer a package of services to those living with HIV/AIDS and (sometimes) their close family members. There are two major ethical challenges. First, the rollout of greater access is gradual, and this means that there is not enough treatment and services for all those who stand to benefit from it. In short, treatment and services in the short term have to be rationed, with the ethical choices that rationing involves[35-37]. Second, when an HIV-positive person and his/her family receive treatment and services, this may only reach the tip of the iceberg. HIV/AIDS is one – and perhaps not the most pressing – of the health-related needs of the program's beneficiaries. HIV-positive persons in low-income countries are vulnerable in many ways: they may suffer from other health conditions for which there is no local or affordable treatment, like cancer or mental illness; their homes may be destroyed by natural disasters or civil strife; they may be children orphaned from their dead parents, or have to take care of such children; and they may have more regular access to antiretroviral treatment than they do to the food that helps them absorb it. Anecdotal reports of AIDS patients selling their drugs to buy other medications for their family members or for food indicate that there may be differences in perception about health-related needs and priorities on the part of global initiatives and local communities. Programs with the goal of reducing global health inequality by providing antiretroviral treatment must decide to what extent they can (or are allowed by their funders to) tackle these other needs. Such decisions regarding 'ancillary care' in biomedical research always have ethical dimensions and implications[38-40]. In Kinshasa, the members of CIBAF have established a monthly ethics session with local research teams, and problems of ancillary care responsibilities are highly prominent in these discussions.

#### Changing health policies

Health policies, in general, aim to promote health by legislating approaches to health prevention, treatment and care. The general assumption is that health policies are not just words on paper, but can have a real impact – especially when integrated into institutional procedures and/or backed by the force of law – on how health interventions are implemented in the real world. Health policies may have a stronger or weaker evidence base, or more or less appropriate in a given context, but that they can have an impact on health is hard to seriously doubt. Altering health policies is therefore another important means of reducing global health inequalities, though again, such changes can raise a network of ethical concerns and challenges. For example, take recent changes in HIV testing policy. For decades, voluntary testing and counseling (VCT) was the model for HIV testing policy around the world. According to the policy as initially promoted, VCT centers should be established that offer intensive pre- and post-HIV test counseling for those who come to these centers to learn about their HIV-status. This policy, stressing individual choice and confidentiality of results, was quite different from past policies regarding other serious infectious diseases, and was likely shaped by the fact that HIV first emerged among stigmatized populations (gay men and injection drug users) who, because there were no effective drugs yet, could not be treated once diagnosed with HIV. With roughly 90% of HIV positive persons in sub-Saharan Africa unaware of their HIV-status, and increasing access to antiretroviral treatment, the World Health Organization and the Centers for Disease Control and Prevention now promote what they call 'provider-initiated' HIV testing policies. One such policy is 'opt-out' HIV testing, whereby patients at clinics and hospitals are told by staff that they will be tested for HIV unless they explicitly decline testing. While the policy has the worthwhile goal of increasing the numbers of persons with knowledge of their HIV status, there are ethical concerns when the policy is promoted in low-income countries, such as the DRC. For example, it is unclear to what extent patients (particularly women) in these countries are capable of declining testing or whether the existence of an 'opt-out' testing policy will lead people to avoid health clinics[41,42]. The weighing of the burdens and benefits of 'provider initiated' testing is also more complicated when those tested are not guaranteed access to HIV treatment[43]. As studies are conducted on the impact of the new HIV testing policies, and treatment access increases, at least some of these ethical concerns may be identified and addressed[44-46]. Again, the point here is not to argue for or against new changes in HIV testing policy, but simply to indicate how changes in health policy trigger complex ethical challenges in low-resource settings that bioethicists, together with other stakeholders involved in the improvement of health in developing countries, must reckon with.

#### Strengthening health care infrastructure

Raising the standards of medical care in low-income countries would go some way in reducing global health inequalities. That health care services in some low-income countries are less than adequate is well-known, and there is growing acknowledgement that it is important to strengthen local health infrastructure, in terms of medical materials and supplies, organization and records-keeping, as well as human resources. In regard to the latter, there is a phenomenon one might call the 'brain-drain capacity-building conundrum.' On the one hand, low-income countries (particularly sub-Saharan Africa) have the lowest nurse-patient and doctor-patient ratios in the world, and therefore there is a great and urgent need for capacity-building of health care professionals in these countries. On the other hand, those training at institutions in low-income countries are increasingly being lured by employment opportunities in more affluent countries, whose demand for health care professionals is increasing as their population ages. For example, out of an estimated 4000 nurses in active service in Malawi in 2005, 453 nurses who had been trained in Malawi were reported to be working in developed countries (mostly United Kingdom), representing 11.3% of the number of the active nurse work force at the time[47].

There is additionally the internal brain drain, i.e. health professionals abandoning the public health sector for more lucrative and career-enhancing positions in the private health sector, foreign-funded health research projects or medical positions within non-governmental organizations. There is an ethical conflict between an individual's right to seek better occupational circumstances for him- or herself and responsibilities towards patients and supportive institutions in his/her country of origin and training. Given this conundrum, those attempting to build capacity among health professionals in low-income countries (including our own efforts in the DRC) must ensure that they are not contributing to the brain drain and making a bad situation even worse. The 'push', 'pull' and 'grab' factors driving the brain drain[48,49], however, must also be tackled at another level, factors having to do with economic conditions in a global market, government policy and international relations.

#### Tackling upstream factors

Debates about globalization have entered the domain of public health with the acknowledgment that forces, transcending the jurisdiction or control of any particular country, profoundly impact on the health of populations. These transnational forces may be responsible for some of the inequalities in health worldwide. Some of these forces include: mass migration due to wars; the international arms and drug trades; the traffic in persons, including women in the sex industry; international trade agreements and policies; climate change, and its effects on disease emergence, prevalence and distribution; international priorities in research and development, including the large proportion spent on military research; debt relationships between high- and low-income nations; unsustainable growth in consumption in high-income countries and population in low-income ones; aggressive advertizing of tobacco by multinational corporations in low-income countries; changes in diets due to increasing trade in foods high in fat and sugar; ecological destruction by extractive industries[50]. Take for example the current global food crisis. Global prices of basic foodstuffs have increased significantly, plunging an estimated additional 100 million people into extreme poverty, and the effects of the food crisis on health will be disproportionately felt in low-income countries. The complex of man-made factors behind acute and chronic global health crises should be critically investigated from a perspective integrating public health research, bioethics, political economy, and history.

Those involved in governance, civil society, public health and bioethics can contribute in different ways to the goal of reducing global health inequalities by tackling these upstream forces. Citizens can press their own governments, through collective action and mobilization, to fulfill their obligations of creating the social, economic and political conditions for greater equality in health. Global institutions, such as the United Nations, International Monetary Fund, and the World Bank, have similar obligations in regard to global health inequalities[51,52]. Those in public health can inform political efforts by rigorously exploring the links between upstream factors and population health. Those in bioethics can (for example) articulate core ethical values – expressing what Benatar calls a 'global state of mind' – to guide and evaluate political and public health efforts to reduce gross disparities in health among and within nations[53].

### By way of conclusion: priorities in bioethics

Should global health inequalities, and the ethical issues associated with them, feature more prominently in bioethics discussions? As some point out, bioethicists may balk at the suggestion that topic selection in bioethics be anything other than a matter of personal choice[8]. However, as indicated in the opening sections, one can question how personal these choices really are. The attention of bioethicists (as well as the popular medical) tends to gravitate towards agonizing dilemmas of patients, family members and clinicians at an individual level. High-technology interventions also have a prominent profile in bioethics discussions, and there is something of a bioethics fashion cycle as ethical reflections on newest inventions (e.g. stem cell research, enhancement technologies, facial transplants, gene transfer therapy) replace those that have become less-than-fresh (e.g. dialysis, IVF, life-support). Bioethics also tends to align itself with whatever topics are currently considered scientifically fundable; unsurprisingly, many bioethicists have made the choice of pursuing the ethics of stem cell research, genomics or bioterrorism during the last decade. The focus of bioethicists on novel technologies may be non-accidentally related to the potential market value of such technologies and the interests of public and private funding institutions, which may set money aside to study their ethical implications. This is not to say that such issues are unimportant. The point here is that the objection to the very idea of priority-setting in bioethics is moot when there is already a social and partly market-based *de facto *process of priority-setting in place.

The question is whether it is desirable, or even ethically justified, for bioethics to continue to reflect something like a '90/10' gap, i.e. a situation where 90% of discussions on bioethics in the literature and the popular media may revolve around issues affecting 10% of the world's population. This situation, as mentioned before, seems to be slowly changing and these changes should be encouraged. But it is important to point out that the preoccupations of mainstream bioethics may not even be representative of the range of possible issues within developed countries themselves. Bioethical questions related to urban poverty, drug use, immigration, occupational hazards in the workplace or environmental injustice make only rare appearances in peer-reviewed bioethics journals, course syllabi, and conferences. These areas of scholarship – tightly linked to issues of social justice – may fall below the radar of many bioethicists due to the social, class and racial barriers between many practitioners of bioethics and affected communities. Commonalities exist between bioethical challenges familiar in the low-income countries and those in underserved or marginalized communities within more affluent nations, arising from historical inequities, limited access to health care, racial discrimination, and gender violence. For this reason, greater attention to ethical issues arising from biomedical research, clinical practice and public health interventions 'far away' might have a positive effect on bioethics 'closer to home', potentially expanding the horizons of the field and enhancing its social relevance.

## Competing interests

The authors declare that they have no competing interests.

## Authors' contributions

SR wrote a first rough draft of the manuscript. BM read the first version and made editorial suggestions. After the first peer-review, SR made substantial revisions to the earlier version in close collaboration with BM. They have both read and approved the final version of the manuscript.

## About the authors

Stuart Rennie obtained his PhD in philosophy and MA in anthropology at the University of Leuven, Belgium. He is currently Research Assistant Professor in the Departments of Dental Ecology, Social Medicine and Health Policy and Management at the University of North Carolina at Chapel Hill. He is ethics consultant for CDC-funded Global AIDS Programs in the Democratic Republic of Congo and Madagascar, and is currently conducting qualitative research on community attitudes towards male circumcision as HIV prevention strategy in Malawi.

Bavon Mupenda has a Master's in Sociology from the University of Lubumbashi (Democratic Republic of Congo) and a MPH in Community Health and Development from the Tropical Institute of Community Health and Development (TICH) in Kenya. A former Fogarty bioethics trainee at Johns Hopkins University, Mr. Mupenda is currently Project Coordinator at the Centre Interdisciplinaire de Bioéthique pour L'Afrique Francophone (CIBAF) and is completing his PhD on prevention of HIV transmission among HIV-positive youth in Kinshasa.
